# Spindle cell lesions of the breast: a diagnostic approach

**DOI:** 10.1007/s00428-021-03162-x

**Published:** 2021-07-29

**Authors:** Emad A. Rakha, Edi Brogi, Isabella Castellano, Cecily Quinn

**Affiliations:** 1 Division of Cancer and Stem Cells, School of Medicine, The University of Nottingham and Nottingham University Hospitals NHS Trust, Nottingham City Hospital, Nottingham, NG5 1PB UK; 2 Department of Pathology At Memorial Sloan Kettering Cancer Center, New York, NY USA; 3 Department of Medical Sciences, University of Turin, Turin, Italy; 4 Histopathology, BreastCheck, Irish National Breast Screening Programme and St. Vincent’s University Hospital, Dublin, Ireland; 5 University College Dublin, Dublin, Ireland

**Keywords:** Breast, Spindle cell lesions, Diagnosis, Immunohistochemistry, Approach

## Abstract

Spindle cell lesions of the breast comprise a heterogeneous group of lesions, ranging from reactive and benign processes to aggressive malignant tumours. Despite their rarity, they attract the attention of breast pathologists due to their overlapping morphological features and diagnostic challenges, particularly on core needle biopsy (CNB) specimens. Pathologists should recognise the wide range of differential diagnoses and be familiar with the diverse morphological appearances of these lesions to make an accurate diagnosis and to suggest proper management of the patients. Clinical history, immunohistochemistry, and molecular assays are helpful in making a correct diagnosis in morphologically challenging cases. In this review, we present our approach for the diagnosis of breast spindle cell lesions, highlighting the main features of each entity and the potential pitfalls, particularly on CNB. Breast spindle cell lesions are generally classified into two main categories: bland-appearing and malignant-appearing lesions. Each category includes a distinct list of differential diagnoses and a panel of immunohistochemical markers. In bland-appearing lesions, it is important to distinguish fibromatosis-like spindle cell metaplastic breast carcinoma from other benign entities and to distinguish fibromatosis from scar tissue. The malignant-appearing category includes spindle cell metaplastic carcinoma, stroma rich malignant phyllodes tumour, other primary and metastatic malignant spindle cell tumours of the breast, including angiosarcoma and melanoma, and benign mimics such as florid granulation tissue and nodular fasciitis.

## Introduction

The component cells of the breast show a high degree of phenotypic plasticity with multiple lines of differentiation resulting in the diverse morphology that is observed in normal, hyperplastic and neoplastic breast tissue [1–5]*.* The origin of spindle cell lesions of the breast (BSCLs) is highly variable and represents multiple lineages. The proliferation of myoepithelial cells [6] and the stromal cells of the breast may result in the formation of BSCLs. All soft tissue SCLs can occur in the breast and BSCLs may also arise from non-breast specific tissue, including skin, deep fascia, underlying muscle, and bone [7, 8]. Breast carcinoma cells may undergo trans-differentiation with epithelial-mesenchymal transition (EMT) resulting in spindle cell metaplasia of neoplastic epithelial cells mimicking mesenchymal stromal cells. The recognition of their epithelial nature or histogenesis relies on the demonstration of epithelial cell characteristics. This includes the presence of structures that indicate epithelial origin including ductal carcinoma in situ (DCIS), invasive breast carcinoma (IBC), no special type (NST), special type, or malignant squamous components, and/or demonstration of epithelial marker expression, including cytokeratin (CK), E-cadherin, and MUC1, on immunohistochemistry (IHC) [9, 10]. Malignant BSCLs that are positive for CK IHC are categorised as invasive carcinomas (metaplastic, “mesenchymal-like”, spindle cell carcinoma). Metastatic tumours to the breast may also assume a spindle cell morphology.

BSCLs encompass a broad range of pathological entities that may be benign, locally aggressive, or malignant. Accurate diagnosis is of crucial importance to ensure appropriate management. The morphological overlap between some of these lesions can lead to misinterpretation or misdiagnosis of benign and malignant entities, particularly in the limited material present in a core needle biopsy (CNB). Ancillary techniques including IHC and molecular assays are often helpful in such cases. Pathologists need to be familiar with the diverse morphological appearances of the different entities, the range of differential diagnoses, and the optimal IHC panels in the various scenarios (Table 1). In this review, we present our approach for the evaluation of BSCLs, highlighting the main features of each entity and the potential pitfalls particularly on CNB. BSCLs can be classified according to history/presentation, lesion size, component cells, interstitial matrix, margin configuration, IHC profile, molecular alterations, or clinical behaviour. We advocate the classification of BSCLs into bland-appearing and malignant-appearing lesions, according to the cytomorphological features of the component cells, with a differential diagnosis and IHC approach for each group that takes into account the morphological overlap and includes both benign and malignant entities [11]. For example, in the group of bland-appearing lesions, it is important to distinguish fibromatosis-like spindle cell metaplastic carcinoma (MBC) from benign entities. The malignant-appearing lesions category includes spindle cell metaplastic carcinoma, stroma rich malignant phyllodes tumour (PT), other primary malignancies e.g. angiosarcoma, metastatic malignant spindle cell tumours of the breast e.g. melanoma and non-malignant entities including nodular fasciitis and florid granulation tissue.

**Table 1:** Criteria that should be considered when diagnosing breast spindle cell lesions (BSCLs)

Criteria	Features
Component cells	- BSCLs may be exclusively composed of spindle cells, or of spindle cells admixed with other cells, including epithelioid cells, adipocytes, inflammatory cells, and muscle cells - Adipose tissue within a BSCL may be a component of the lesion, as in spindle cell lipoma and myofibroblastoma (morphologically similar lesions; spindle cell lipoma is rare and lacks ER, desmin and actin immunoreactivity), or represent entrapped fat cells resulting from neoplastic infiltration by the lesion, as seen at the periphery of infiltrative lesions - Similarly, epithelial components may represent entrapped normal mammary epithelium as in fibromatosis, be a distinct component of the lesion as in fibroepithelial lesions or be part of the spindle cell proliferative process as in MBC - A benign epithelial component may be seen in both benign and malignant BSCLs; however, a malignant epithelial component denotes a malignant lesion
Cytonuclear atypia	- High-grade cytonuclear atypia such as nuclear pleomorphism, hyperchromatism, prominent nucleoli and irregular nuclear outlines indicate a malignant process - In contrast, absent or low-grade atypia may represent a reactive, benign, or low-grade malignant process
Mitosis	- Frequent mitotic figures, including atypical forms, are a feature of high-grade malignancy - Although some reactive BSCLs, such as nodular fasciitis, may show frequent mitotic figures, these are typically of normal form and not associated with cytonuclear atypia
Cellularity	- Low, moderate, or high cellularity as assessed by nuclear spacing, nuclear touching and nuclear overlapping
Growth pattern	- Variable including fascicular, storiform, diffuse or whorled
Breast parenchyma	- BSCLs may contain glandular parenchymal elements (acini and ducts) as entrapped tissue as a component of the lesion or may be devoid of such elements
Margins	- Infiltrating or well defined as evidenced by regularity of the lesion border, entrapment of fat cells at the periphery of the lesion or infiltration of the surrounding breast parenchymal tissue and muscle
Immunohistochemistry	- IHC is very helpful in many scenarios in which BSCLs show overlapping morphology - The choice of markers should be based on the differential diagnosis - Biomarkers indicating epithelial differentiation are particularly important in the diagnosis of bland-appearing BSCLs and sub-classification of malignant BSCLs
Necrosis	- Necrosis is usually seen in high-grade malignant lesions. Necrosis should be differentiated from infarction
Other features	- The presence of *in-situ* carcinoma or a conventional mammary-type invasive breast carcinoma component favours the diagnosis of MBC - The presence of thick bundles of hyalinized (‘ropy’) collagen between the spindle cells is an important feature of myofibroblastoma - Diffuse infiltration of the surrounding tissue and irregular margins are features of fibromatosis and of malignant lesions - Patient age, lesion size, location relative to skin and deep structures and rate of growth are important features in considering a diagnosis

*BSCL* breast spindle cell lesion, *MBC* metaplastic breast carcinoma, *IHC* immunohistochemistry

## Bland-appearing spindle cell lesions

### Fibromatosis-like metaplastic breast carcinoma

**Fig. 1:**
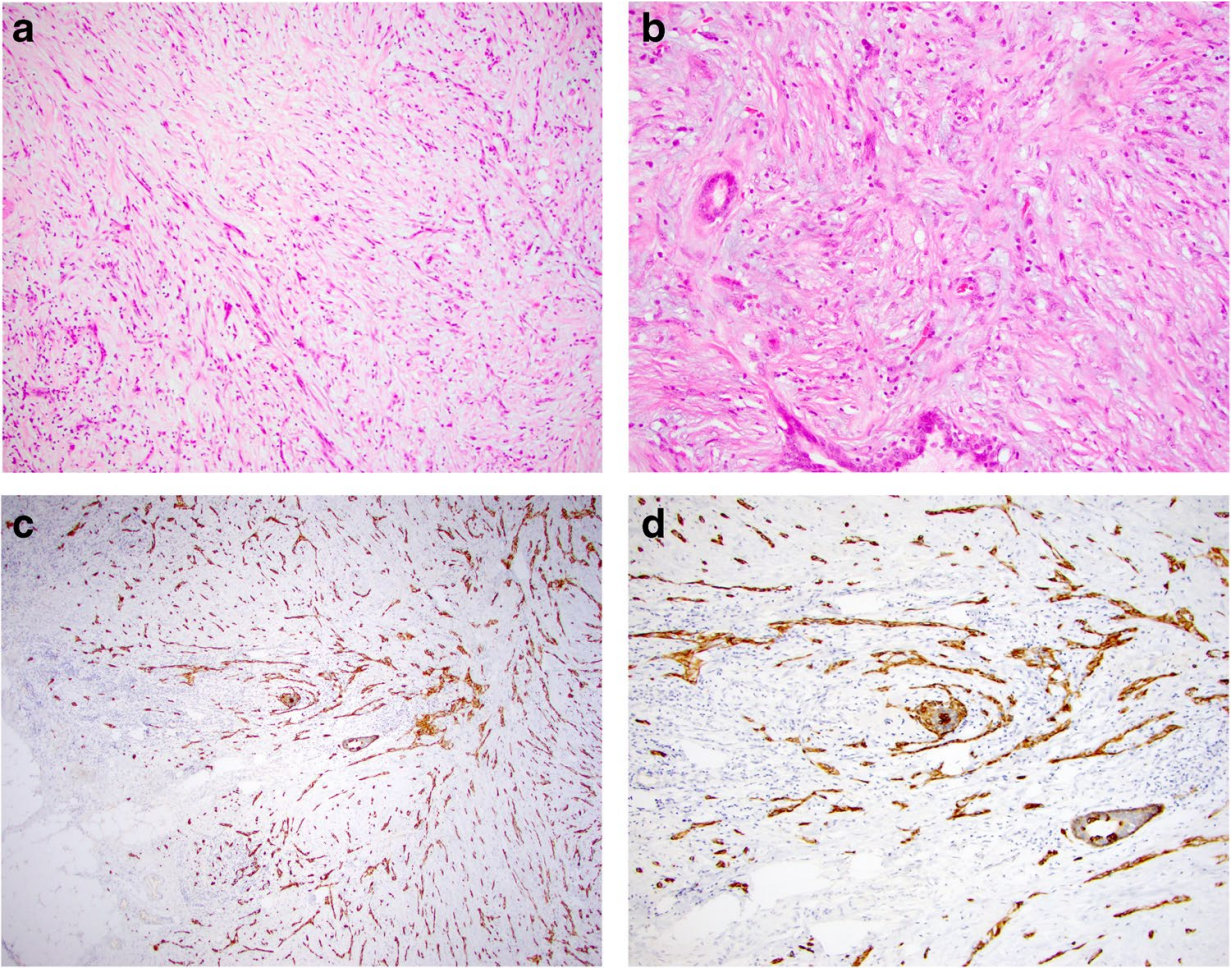
Low-grade fibromatosis-like metaplastic breast carcinoma on H&E (**a**,** b**). Cytokeratin (34betaE12) immunohistochemistry highlights the malignant epithelial cells (**c**, **d**)

The most important challenge in the diagnosis of bland-appearing BSCLs is the exclusion of fibromatosis-like MBC [11–14] (Table 2). This rare tumour shows the unusual combination of spindle cell metaplasia of malignant breast epithelial cells and bland cytological features with minimal nuclear pleomorphism and scarce mitotic activity. Distinction from fibromatosis and other bland-appearing BSCLs may be difficult particularly on CNB also due to similar growth pattern on imaging. These tumours typically lack an in situ component and do not show other features of conventional type low-grade IBC. Regressive changes including stromal fibrosis, sclerosis, collagenisation, and inflammatory cell infiltrate with lymphoid follicles are commonly seen. Fibromatosis-like MBC may be associated with sclerosing lesions such as papillary lesions or radial scars, making diagnosis even more challenging, particularly in the early stages of evolution. This entity is one of the rare breast lesions in which a diagnosis of malignancy may rely solely on the demonstration of CK expression on IHC despite the absence of definite morphological features of malignancy (Fig. 1). In contrast to fibromatosis-like MBC, low-/intermediate-grade spindle cell MBC displays cytological atypia sufficient for a diagnosis of malignancy and distinction from fibromatosis. In low-grade adenosquamous MBC, the neoplastic elements comprise tubules, epithelioid clusters, and squamous structures with distinct morphology and immunoprofile whereas the accompanying spindle cell population is typically reactive stromal/fibroblastic type. Dwyer and Clark [12] indicated that squamous or glandular epithelial elements may be seen in fibromatosis-like MBC but these should be a minor component accounting for less than 5% of the total tumour area.

Although fibromatosis is a locally aggressive lesion that was traditionally excised after CNB diagnosis, the current trend is to adopt a more conservative observational approach [13, 15]. Surgical excision is associated with a high rate of re-excision (33–46%) and the rate of local recurrence (9–15%) is not significantly lower than in the active surveillance group (watch-and-wait approach; 12%) [13, 14]. Therefore, the distinction of fibromatosis from fibromatosis-like MBC, particularly on CNB, is of crucial importance. Recognition of focal cytological atypia and areas of epithelioid differentiation on morphology (e.g. plump cells with slightly abundant cytoplasm arranged singly or in small clusters) or on IHC assist accurate diagnosis of fibromatosis-like MBC. Although not specific, a prominent inflammatory cell infiltrate with lymphoid follicles may be seen. We have also observed that nuclear spacing, observed in the long fascicles of fibromatosis, is typically lacking in fibromatosis-like MBC. It is our experience that breast fibromatosis does not show cytological atypia and a bland-appearing spindle cell lesion with even a mild degree of cytological atypia, particularly with dark hyperchromatic pleomorphic nuclei, should raise the suspicion of fibromatosis-like MBC. Pleomorphic “open face” nuclei can be seen in nodular fasciitis, but other features help to accurately distinguish this from other lesions.

Although fibromatosis-like MBC is associated with an excellent prognosis as compared with other MBCs, [16, 17] local recurrence may occur and occasional metastases have been reported [18]. In the study by Gobbi et al. [16], 8 of 18 patients with fibromatosis-like MBC, with available clinical follow-up, developed local recurrence. Seven of these 8 patients had been treated with excisional biopsy alone with no statistically significant difference in outcome identified in the recurrent tumour group compared to patients with non-recurrent tumours. Therefore, these authors concluded that recurrences were likely to be directly related to inadequate local excision. In our practice, we have observed, on review, that many large-sized spindle cell MBCs diagnosed as fibromatosis-like MBC contained foci of moderate cytological atypia, which may explain the reported metastases in some series [17]. Therefore, it is important to recognise that no definitive conclusion regarding the biological behaviour of fibromatosis-like MBC can be made because most case series are limited by small sample size, the morphological overlap between this entity and other types of spindle cell MBC, variable clinical follow-up intervals, and differences in treatment regimens. Currently, these tumours are managed in accordance with protocols for other low-grade conventional type IBCs and systemic chemotherapy is not recommended for pure early-stage tumours. These tumours have a low potential for lymph node metastasis [18] but sentinel lymph node sampling is required for staging.

The remaining bland-appearing BSCLs can be classified according to the nature or histogenesis of the proliferating cells as follows:

### Lesions of fibroblastic/myofibroblastic origin

These include fibromatosis [19], nodular fasciitis [20–22], scar/reactive spindle cell nodules [23], myofibroblastoma [24], solitary fibrous tumour [25, 26], inflammatory myofibroblastic tumour [27], and cellular pseudoangiomatous stromal hyperplasia (PASH) [28] (Tables 2 and 3).

**Fig. 2:**
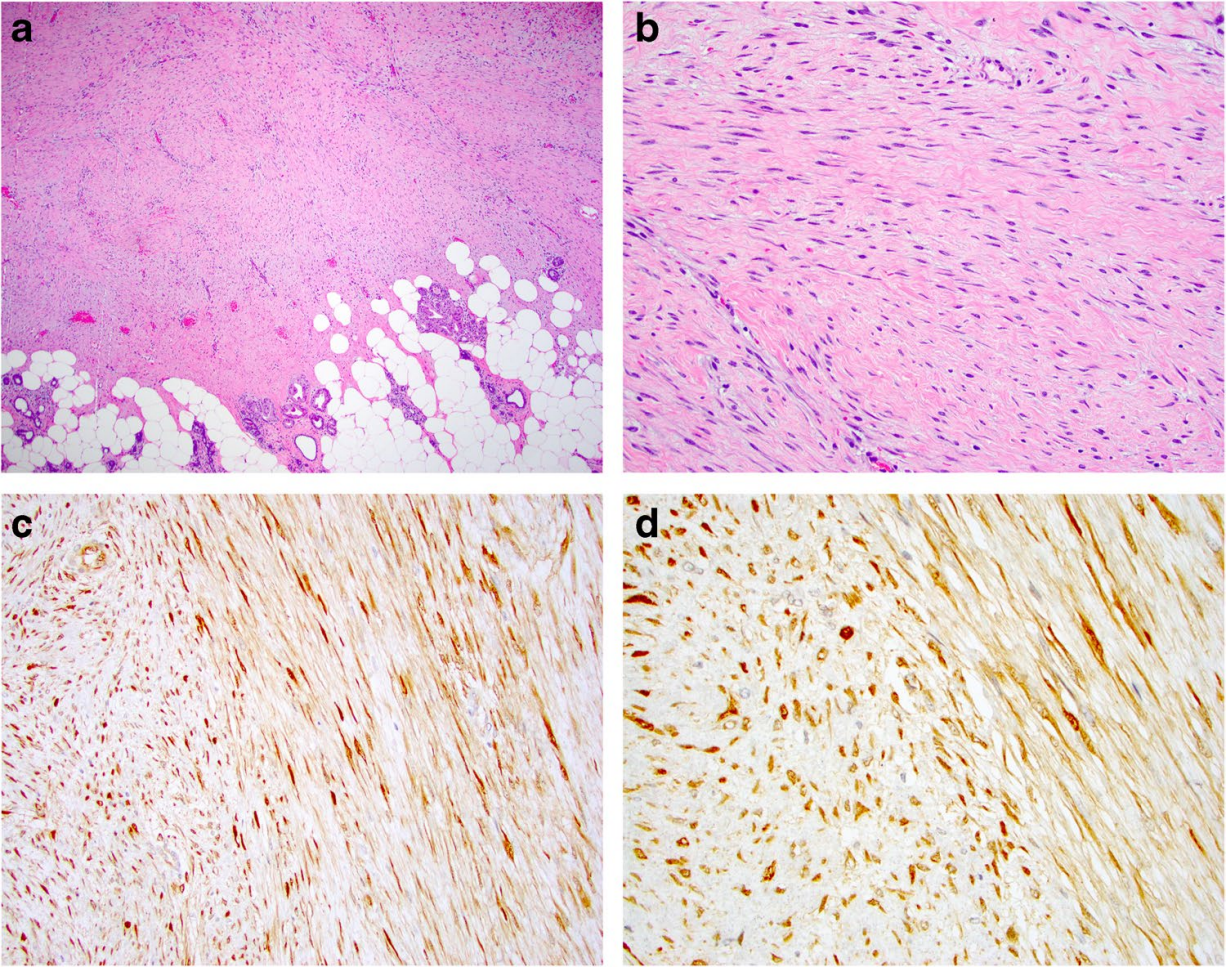
Fibromatosis featuring an infiltrative margin (**a**) with bland appearing spindle cells showing spacing of nuclei (**b**). Beta-catenin staining shows nuclear positivity (**c**, **d**)

*Fibromatosis* of the breast is a rare locally aggressive or benign mesenchymal transformation of connective tissue origin, usually originating from the fascia of pectoral muscles or Cooper’s ligaments [19, 29–32]. The distinction of fibromatosis from fibromatosis-like MBC or other BSCLs of the breast is difficult on clinical and radiological examination and histologic assessment is essential for a definite diagnosis. Although breast fibromatosis is typically diffusely infiltrative with entrapped fat at the periphery, the lesional cell nuclei are bland and characteristically spaced (Fig. 2). Lymphocytes are often present at the periphery. On IHC, fibromatosis cells typically display β-catenin nuclear staining and smooth muscle actin (SMA) cytoplasmic staining and lack immunoreactivity of CKs, p63, and CD34. Although β-catenin nuclear staining is a characteristic feature of fibromatosis, focal weak nuclear staining of β-catenin may be seen in some (23%) spindle cell MBCs [33].

**Fig. 3:**
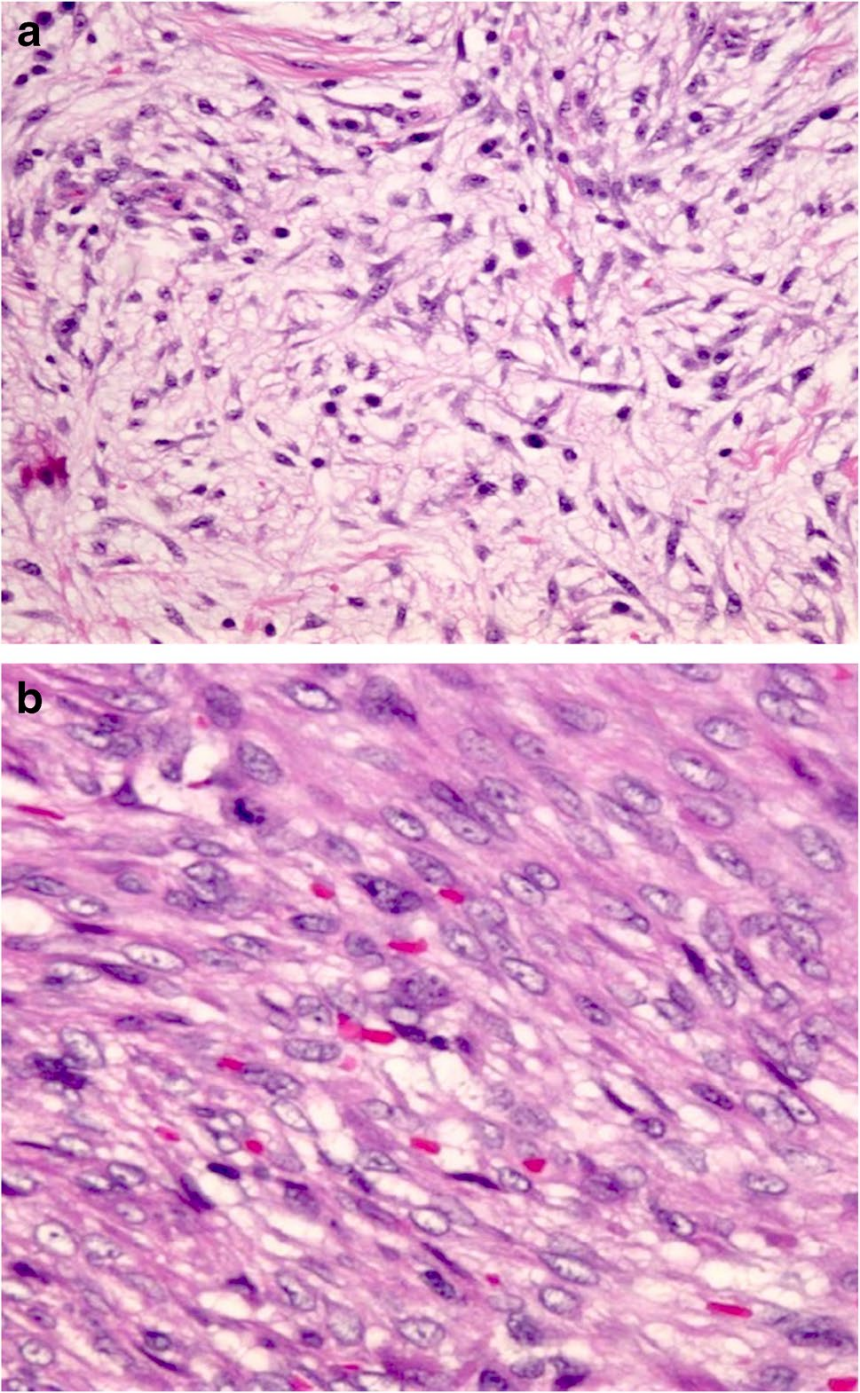
Nodular fasciitis showing a tissue culture like pattern with loose stroma (**a**). Extravasation of red blood cells and mitotic figures may also be seen (**b**)

*Nodular fasciitis* is a rapidly growing reactive/benign self-limiting mass-forming breast lesion that may be tender or painful. It is composed of a clonal proliferation of bland-appearing stellate fibroblasts arranged in a loose fascicular to a storiform pattern (tissue culture-like or feathery growth pattern) (Fig. 3). The cellularity is variable and the extracellular matrix ranges from myxoid to collagenous. Extravasated red blood cells are often present, and mitoses may be frequent but without abnormal forms. Nodular fasciitis can be differentiated from spindle cell MBC by its characteristic clinical and histological features and by IHC which demonstrates actin positivity and lack CKs and p63 immunoreactivity [11].

**Fig. 4:**
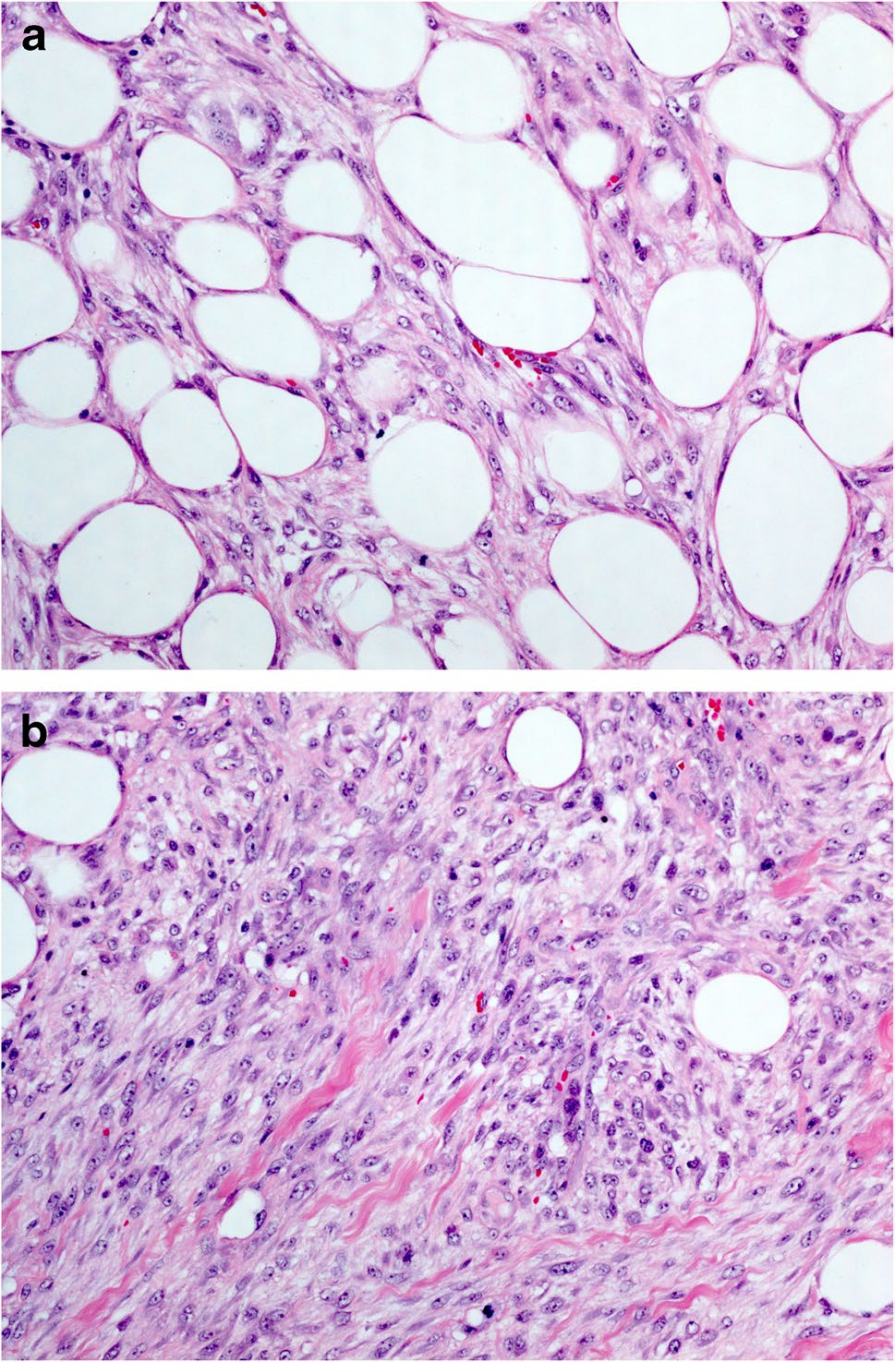
Tissue reaction to trauma resulting from previous surgery with fat necrosis and a florid histiocytic and myofibroblastic reaction (**a**). Florid reactive changes and mitotic figures may be seen (**b**) but inflammatory cells, hemosiderin deposition and occasional multinucleated giant cells are usually also present

*Scar* is one of the most common bland-appearing BSCLs of the breast and accurate identification is facilitated by a history of trauma or previous surgery and recognition of additional accompanying features including fat necrosis, haemosiderin deposition, foamy macrophages, and foreign body giant cells. A reactive spindle cell nodule is likely to represent an exuberant reparative process (i.e. young scar) (Fig. 4). It may reach a large size and be associated with fibro-sclerotic breast lesions. Some mature scars mimic fibromatosis or fibromatosis-like MBC, particularly on CNB. In doubtful cases, lack of expression of CKs, p63, CD34, and nuclear β-catenin on IHC helps to confirm a diagnosis of a scar. Scar may express SMA and have a similar IHC profile to nodular fasciitis. The latter is distinguished by its typical clinical history of rapid growth, superficial location, and characteristic morphology.

**Fig. 5:**
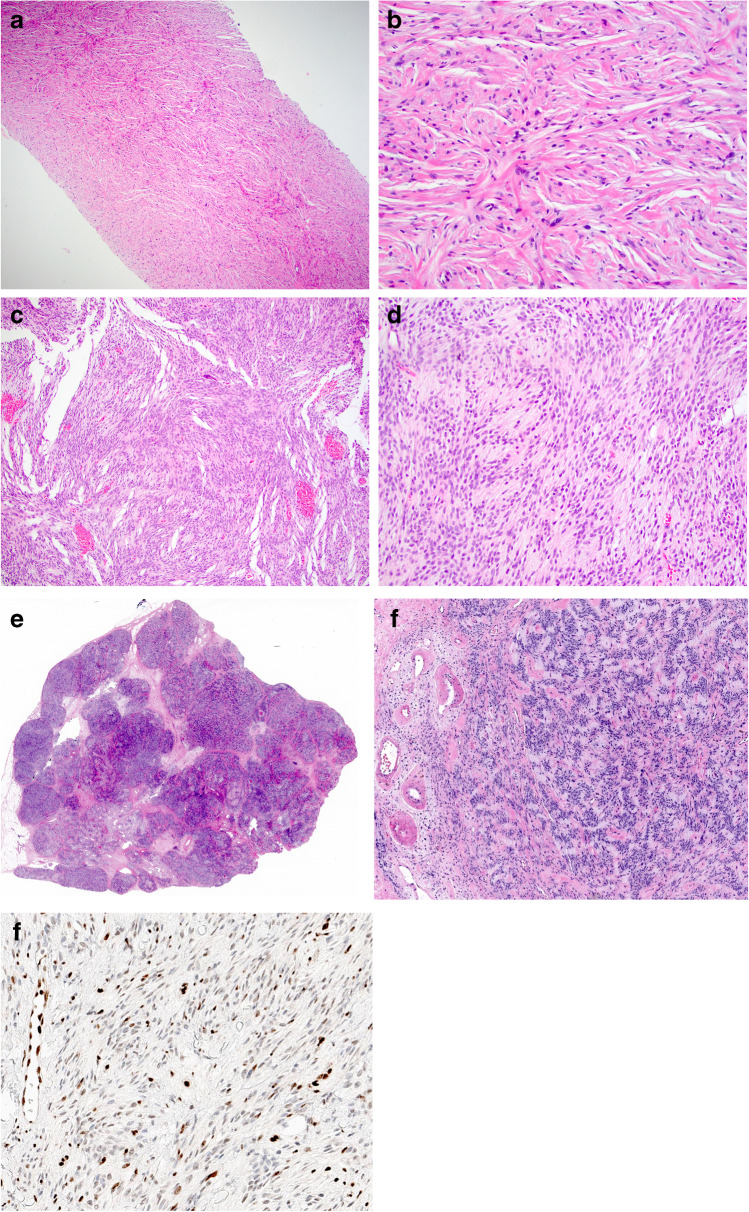
Myofibroblastoma: A needle core biopsy (**a**) with higher power view (**b**) showing bland looking spindle cells with intervening thick hyalinised collagen bands. Excision specimen of myofibroblastoma showing increased cellularity and ovoid nuclei (**c**, **d**). **e** and **f** show an example of myofibroblastoma with palisaded nuclei. **g** shows loss of nuclear expression of Rb gene on immunohistochemistry

*Myofibroblastoma* of the breast, a tumour showing myofibroblastic differentiation without epithelial features, may simulate spindle cell MBC and other BSCLs of the breast [34]. It has been described mainly in men; however, this lesion occurs in women as well. The morphology varies but typically shows fascicular growth of spindle cells with bands of intervening collagen fibres, devoid of breast ducts and lobules (Fig. 5). It may be cellular, shows amianthoid fibers, and often contains a variable adipocytic component. The combination of ER, CD34, SMA, and desmin expression and loss of Rb expression is characteristic of myofibroblastoma. PR, CD10, CD99, and Bcl2 may also be positive. Unlike spindle cell MBC, myofibroblastoma lacks expression of p63 and CKs [11].

**Fig. 6:**
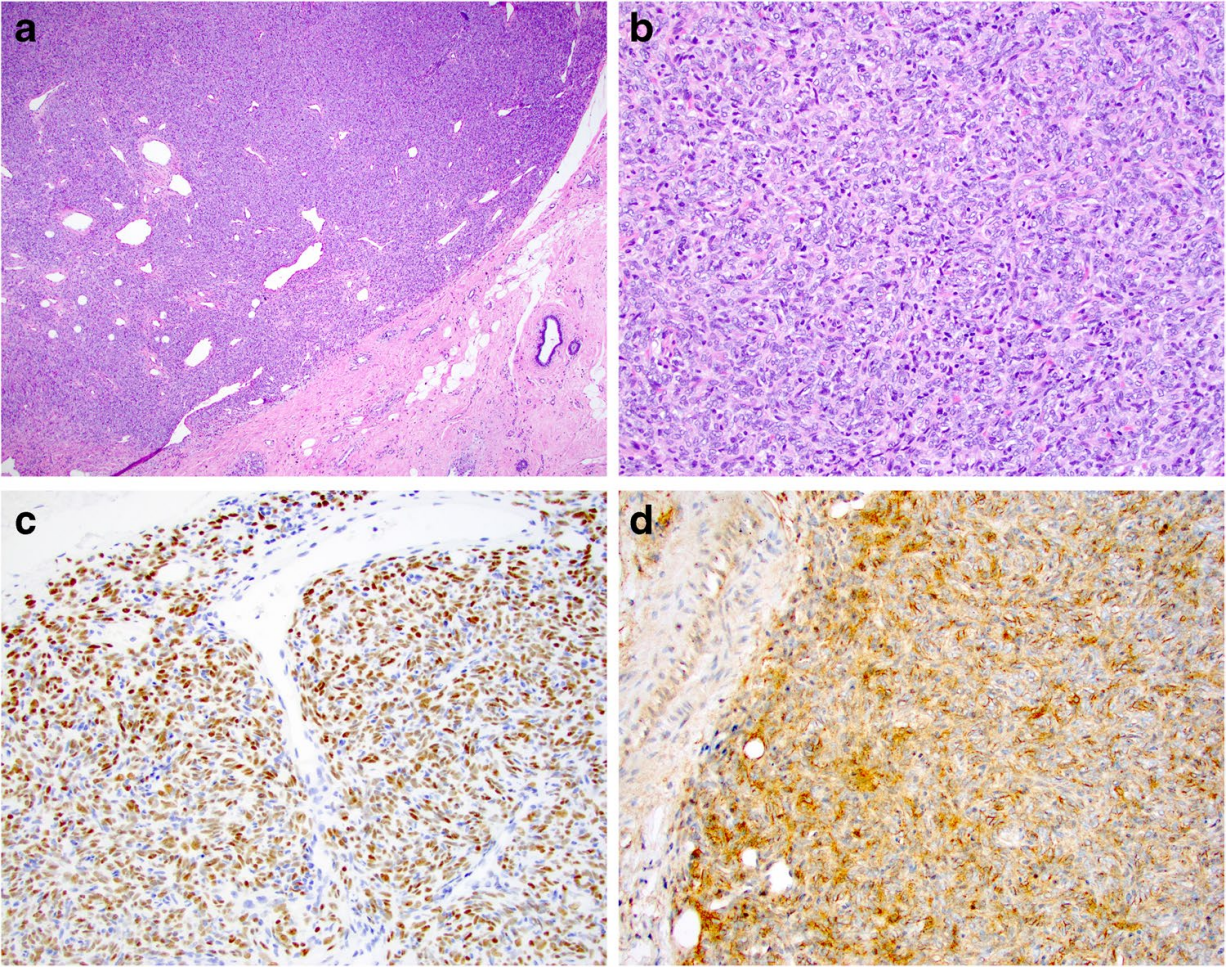
A case of solitary fibrous tumour (SFT) with a staghorn-like vasculature and a featureless pattern in which spindle cells and collagen bundles are randomly dispersed throughout the tumour (**a**). The cells are ovoid to fusiform and spindle-shaped with indistinct cell borders (**b**) arranged haphazardly or in short, ill-defined fascicles. Immunohistochemistry shows nuclear staining of STAT6 (**c**) and cytoplasmic staining of CD99 (**d**)

*Solitary fibrous tumour (SFT)* is a rare BSCL of uncertain origin that tends to retain its typical morphology. It is variably cellular and is composed of round to spindle-shaped cells with little cytoplasm, often arranged in a short storiform pattern (patternless distribution), traversed by eosinophilic bands of collagen, and thin-walled branching blood vessels (Fig. 6). SFT is characterised by diffuse and strong IHC expression of STAT6 [35], in addition to CD34, CD99, and Bcl2.

*Inflammatory myofibroblastic* *tumour* of the breast is distinct from post-operative myofibroblastic repair reaction and traumatic fat necrosis which may also be mistaken for spindle cell MBC [11, 36]. It usually presents as a painless, circumscribed, firm mass. CK positivity, particularly on CNB, may lead to an erroneous diagnosis of spindle cell MBC. However, inflammatory myofibroblastic tumour has a significant inflammatory component dominated by plasma cells intermingled with fascicles of bland-appearing spindle cells with eosinophilic cytoplasm and ovoid or tapering nuclei. IHC may also show expression of SMA and ALK1 is positive in approximately 60% of cases. p63, desmin, S100, and CD34 are negative.

**Fig. 7:**
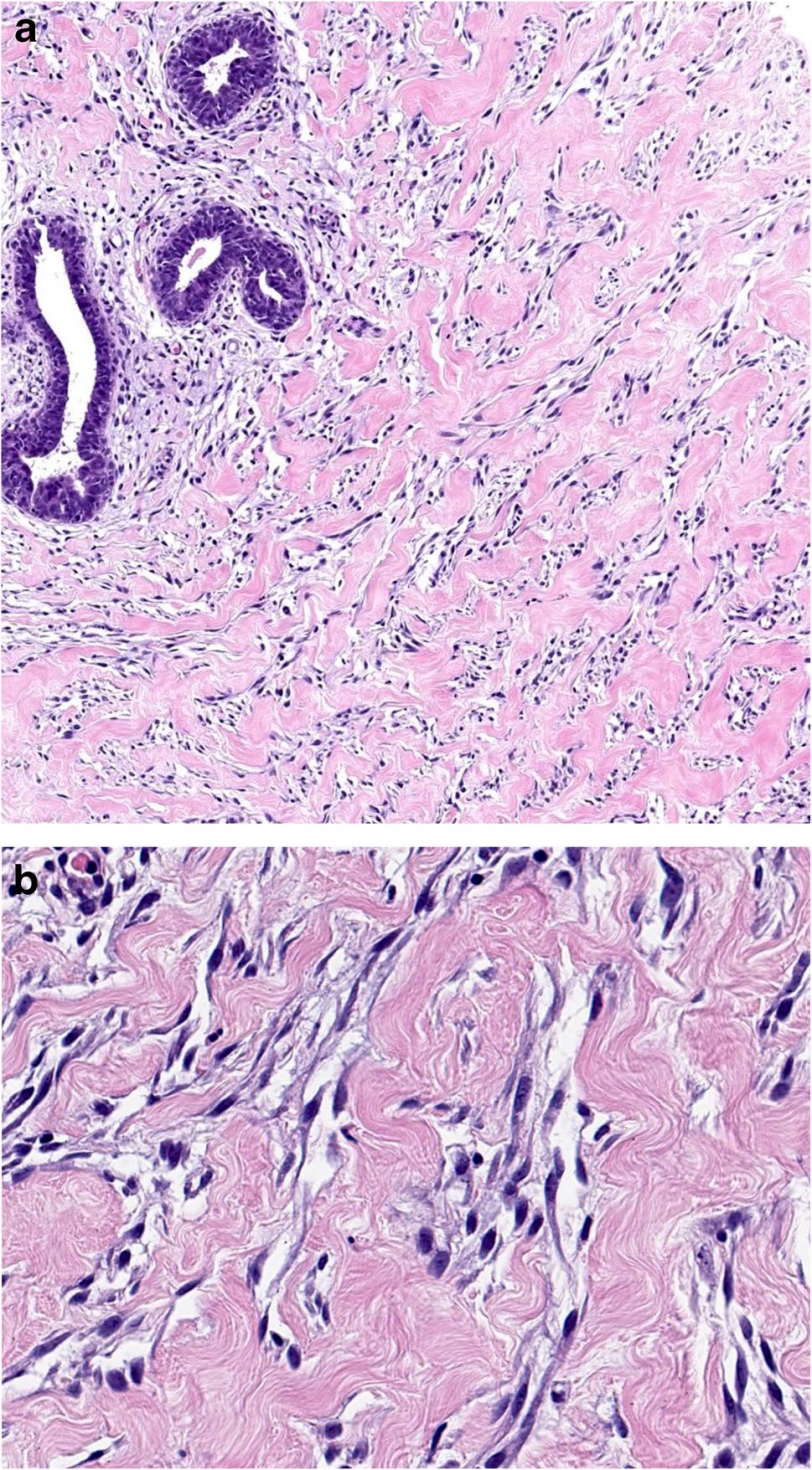
Pseudoangiomatous stromal hyperplasia (PASH) shows complex inter-anastomosing spaces in dense collagenous stroma (**a**, **b**). The spindle-shaped myofibroblasts lining the slit-like spaces simulate endothelial cells. Spaces are usually empty but may contain rare red blood cells. Some cases may be cellular with plump spindle cells, which may obscure the pseudoangiomatous structure

*Cellular PASH* is rare and is typically positive for CD34 and PR and is variably positive for actin and desmin. It displays the characteristic pseudo-vascular channels, at least focally, lined by spindle-shaped myofibroblasts which simulate endothelial cells (Fig. 7) but are negative for other endothelial cell markers including CD31 and D2-40 [37].

### Lesions of myoepithelial cell origin

Adenomyoepithelioma with a spindle cell pattern and cellular spindle forms of sclerosing adenosis display characteristic morphology and are usually diagnosed on H&E. In doubtful cases, IHC highlights the dual epithelial and myoepithelial cell population and assists diagnosis.

### Lesions of intralobular stromal cell origin

Although usually of borderline or malignant nature, stroma-rich phyllodes tumour (PT) may present a bland cytological appearance and is included in the differential diagnosis of bland-appearing BSCLs. Recognition of epithelial clefts may facilitate diagnosis. It is our experience that stroma-rich PT with a bland SCL appearance is typical CD34 positive. CD34-negative stroma is usually seen in high-grade PT.

### Lesions of smooth muscle origin

Leiomyoma and low-grade leiomyosarcoma may arise from the smooth muscle of the breast^24^. They usually maintain characteristic morphology and IHC evidence of smooth muscle differentiation, strongly positive for smooth muscle markers and negative for CD34, p63, and CKs. Leiomyoma usually involves the nipple region but is occasionally seen within the breast parenchyma.

### Lesions of nerve sheath origin

Neurofibroma and schwannoma may rarely occur in the breast and are diagnosed on morphology with IHC if required.

### Other BSCLs

Some rare examples of hamartoma show prominent myoid differentiation that may result in spindle cell morphology. Spindle cell and atypical lipomas that can occur in the breast may rarely simulate other spindle cell tumours. Cellular variants of angiolipoma of the breast may simulate spindle cell MBC and even angiosarcoma [38, 39].

Although low-grade angiosarcoma, dermatofibrosarcoma protuberans (DFSP) and low-grade fibromyxoid sarcoma, and myofibroblastic sarcoma typically show bland cytological features, these tumours show distinct architectural features sufficient for their identification.

#### Immunohistochemistry

IHC is a useful ancillary tool in the diagnosis of bland-appearing BSCLs as outlined above. It is best to use a panel of antibodies, to maintain a broad differential diagnosis and to interpret the findings in the light of morphology and clinical context. No IHC marker is completely sensitive or specific and pathologist should not attach undue significance to any individual marker. When spindle cell MBC is in the differential diagnosis, it is important to use a panel of antibodies against epithelial markers, including both low and high molecular weight CKs as no individual antibody stains all MBCs [10, 40]. Most fibromatosis-like MBCs show positivity for high molecular weight and broad-spectrum CKs and p63. Some may show only show focal (or no) immunoreactivity for CKs, but these variants often express p63. Myoepithelial/myoid markers including SMA and smooth muscle myosin heavy change (SMMHC), and vimentin may be positive in MBC so a diagnosis of MBC is not excluded but may help to differentiate other lesions (Tables 2 and 3). Nuclear β-catenin is observed in most fibromatoses but may be seen in some MBCS which should be excluded using CK and p63 IHC. CD34 is negative in fibromatosis-like MBC and fibromatosis but is typically positive in PASH, stroma-rich PT, myofibroblastoma, and solitary fibrous tumour.

**Table 2:** Most common bland-appearing spindle cell lesions of the breast and their key features

Lesion	Key features	Other features
Fibromatosis-like metaplastic breast carcinoma (MBC)	Infiltrative, focal mild atypia and focal epithelioid differentiation	- Low-grade spindle cell proliferation with variable cellularity - Cells with pale eosinophilic cytoplasm and slender nuclei with tapered edges and finely distributed chromatin admixed with plump fusiform and polygonal tumour cells that have more rounded nuclei and are arranged in”epithelioid “ clumps mainly seen centrally in the tumour - Infiltrative with entrapped normal breast structures - Regressive changes: collagenisation, scattered inflammatory cell infiltrate comprised of lymphocytes and plasma cells with occasional lymphoid follicles at the edges of the tumour. May be associated with papillary lesion or radial scar - IHC expression of CKs and/or p63 (and p40). CK + cells are seen in almost all cases and usually appear as cords or sheets of polygonal cells, rarely as isolated positive cells. SMA is often positive particularly in CK negative cells. Typically negative for CD34, hormone receptors and HER2
Scar	Fat necrosis, haemosiderin-laden macrophages	- History of trauma/procedure and presence of biopsy site-associated changes such as hemosiderin deposition, fat necrosis, foamy macrophages, and foreign body giant cells - Distinction of post-operative scar from residual fibromatosis may be extremely difficult but a fascicular growth pattern and entrapment of breast parenchyma are not usually seen in a scar - Reactive spindle cell nodule is likely to represent an exuberant reparative process (i.e., young scar) but may reach a large size and be associated with breast fibro-sclerotic lesions - IHC: shows positive SMA expression but negative immunoreactivity of β-catenin, desmin and CD34
Fibromatosis	Infiltrative, long fascicles, nuclear spacing	- Locally infiltrative, non-metastatic, lesion frequently arises from deep fascia - Usually solitary non-tender ill-defined mass - May be spiculated on mammography mimicking carcinoma. US and MRI are more sensitive for its detection - Long fascicles; variable collagen deposition and cellularity, diffusely infiltrative with entrapped fat at periphery. The lesion nuclei are characteristically spaced - Lymphocytes are often seen at the periphery - IHC: nuclear staining of β-catenin and cytoplasmic staining of SMA. CD34, CKs, p63, desmin and S100 are negative
Myofibroblastoma	No atypia, devoid of breast parenchyma, ropy collagen	- Benign solitary slowly growing often as a circumscribed tumour - Variable morphology but typically fascicular growth of spindle cells with bands of collagen fibres, amianthoid fibres, variable adipocytic component, may be cellular. No regularly spaced nuclei. Devoid of breast glandular elements - IHC: positive for CD34, desmin, SMA, ER, PR, CD99, BCl2 and CD10
Nodular fasciitis	Rapidly growth lesion with short history, devoid of breast parenchyma, extravasation of RBCs	- Rapidly growing, may be tender or painful, self-limiting mass-forming composed of clonal cellular proliferation - Well-circumscribed; tissue culture-like fibroblasts with plump vesicular nuclei; myxoid stroma; extravasated RBCs. Mitoses may be frequent but no abnormal forms - IHC: positivity for actin

*CK* cytokeratin, *MBC* metaplastic breast carcinoma. Most of the lesions, apart from nodular fasciitis, show rare or no mitoses. Atypia and necrosis are not features of this category of BSCLs. All entities, apart from MBC and some cases of IMT, typically lack cytokeratin immunoreactivity

**Table 3:** Less common bland-appearing spindle cell lesions of the breast and their key features

Lesion	Key features	Other features
Solitary fibrous tumour (SFT)	Patternless pattern and hemangiopericytoma-like vasculature	- Solitary circumscribed firm tumour similar to the more common soft tissue SFT but may overlap with breast myofibroblastoma - Ovoid spindle cells with inconspicuous nucleoli. May show bundles of hyalinised collagen and perivascular inflammatory infiltrate - IHC: Strong nuclear positivity for STAT6, and negative for desmin
Pseudoangiomatous stromal hyperplasia (PASH)	Pseudovascular spaces	- Usually comprises a component of other breast lesions but may present as a pure circumscribed nodular mass. Dominant feature of gynaecomastia - Collagenous stroma separated by slit-like, inter-anastomosing pseudo-vascular spaces lined by spindle cells resembling endothelial cells - IHC: positivity for CD34 and PR, variable positivity for actin and desmin and negative for other endothelial markers
Inflammatory myofibroblastic tumour (IMT)	Inflammatory cells with plasma cells admixed with the spindle cells	- Painless circumscribed firm mass - Significant inflammatory cell component dominated by plasma cells. Fascicles of spindle cells with eosinophilic cytoplasm and ovoid or tapering nuclei - IHC: positive for SMA, ALK may show CK positivity
Benign phyllodes	Epithelial clefts	- Benign PT presenting as SCL without epithelial lined clefts is rare, even on core biopsy
Schwannoma	Wavy nuclei and variable cellularity, Verocay bodies and nuclear palisading	- Painless lump mainly in the skin - May show biphasic pattern with compact hypercellular Antoni A areas and myxoid hypocellular Antoni B areas with characteristic nuclear palisading. Wavy nuclei and ill-defined cytoplasm. Amianthoid fibres or collagenous spherules - IHC: positive for S100 with strong diffuse nuclear and cytoplasmic staining in Schwann cells
Neurofibroma	Neural differentiation	- Non-encapsulated with all elements of peripheral nerves including axons (silver positive). Less of a fascicular pattern than fibromatosis - Unlike schwannoma, no Verocay bodies, nuclear palisading or hyalinised thickening of vessel walls - IHC: positive for S100 that highlights Schwann cells with no staining of the intervening stroma and perineural cells. CD34 + (focal), Factor 13a (focal)
Leiomyoma	Smooth muscle differentiation, often in the nipple	- Slowly growing mass often in sub-areolar area. May be tender or painful. Deep lesions need to be distinguished from other breast lesions with prominent smooth muscle differentiation - Interlacing fascicles of spindle cells with cigar-shaped nuclei and eosinophilic cytoplasm. Atypia and mitotic figures favour the diagnosis of leiomyosarcoma - IHC: positive for SMA, desmin and caldesmon
Spindle cell lipoma	Fat component	- A rare variant of breast lipoma characterised by a mixture of collagen-forming uniform spindle cells and mature fat cells with varying degrees of myxoid change - May contain entrapped breast parenchymal elements - IHC: positive for CD34, and negative for ER, SMA, desmin and S100

*CK* cytokeratin, *MBC* metaplastic breast carcinoma

#### Molecular pathology

Mutations at exon 3 of the β-catenin gene (*CTNNB1*) have been reported in fibromatosis of the breast [41], with a high level of nuclear β-catenin IHC staining identified in > 80% of lesions [41, 42]. These mutations, with the nuclear expression of β-catenin, assist the distinction of breast fibromatosis from morphologically similar reactive and neoplastic processes [43]. Although mutations in *CTNNB1* were not identified in PTs or MBCs [33], nuclear β-catenin is common in benign and malignant PTS and may be seen in up to 23% of MBCs [33]. *CTNNB1* and *APC* (adenomatous polyposis coli) gene mutations are mutually exclusive. *APC* mutations are detected in approximately 11% of breast fibromatosis [44].

Partial monosomy of 13q and 16q with deletion of the 13q14 region, harbouring *RB* and *FKHR*, has been reported in > 50% of myofibroblastomas [45]. Rearrangements affecting 13q and 16q typically occur in spindle cell lipomas [46]. These alterations have not been detected in SFT [47]. In nodular fasciitis, a balanced translocation t(17;22)(p13;q13), resulting in *MYH9–USP6* fusion has been identified [22]. Using copy number analysis, Takano et al. [48] have demonstrated that low-grade fibromatosis-like MBCs are characterised by low genomic instability and do not share copy number aberrations with other types of MBC. They suggest that this entity is a unique tumour subtype with a genotype that reflects its apparent homogeneous morphology and phenotype [48].

## Malignant-appearing breast spindle cell lesions

The most important, and probably the most common, lesion in this category is spindle cell MBC (Table 4). Tumours may be pure SCLs, comprising a mesenchymal-like malignant spindle cell proliferation, or mixed with a squamous or conventional IBC component [49, 50]. The spindle cell component shows a fascicular, storiform, or haphazard growth pattern, usually with an infiltrative border. Cytological atypia is moderate or marked and mitotic figures are usually easily identified. Foci of necrosis may be present. Heterologous elements such as chondrosarcomatous and osteosarcomatous components are seen in some MBCs and may also be seen in malignant PT. In these tumours, additional features including architectural clefting with the formation of cystic spaces, IHC with emphasis on CD34 and cytokeratin expression, and the co-existence of DCIS or IBC are used to differentiate these two entities. In mixed spindle cell MBC, a gradual transition between the spindle cell and epithelioid (adenocarcinomatous) components is an important finding as some tumours lack CK expression in the spindle cell component while retaining it in the epithelioid component. Spindle cell MBCs, pure and mixed, are graded using the Nottingham grading system [51] according to the highest-grade component. They are typically hormone receptor and HER2-neu negative (triple negative). Spindle cell MBCs tend to pursue an aggressive clinical course with high metastatic potential [49].

**Table 4:** Most common malignant-appearing spindle cell lesions of the breast and their key features

Lesion	Key features	Other features
High-grade spindle cell MBC*	Presence of DCIS or another conventional-type invasive breast carcinoma component, presence of squamous cell carcinoma component, If not, positivity of CKs and negativity of CD34	- Classic sarcomatoid / spindle cell metaplastic breast carcinomas show variable combinations of malignant mesenchymal and epithelial tissue - Variable cellularity and architecture (fascicular, storiform but rarely herringbone pattern) - The epithelioid component is frequently poorly differentiated adenocarcinomatous but may be squamous. Associated DCIS is not uncommon - IHC: expression of CKs, which can be focal or diffuse. Large panel of markers should be used as some Cks may be negative. p63 is typically positive in lower grade spindle cell and squamous areas but can be positive in high-grade spindle cell areas. Variable degree of positivity for myoepithelial markers. Typically negative for CD34, hormone receptor and HER2
Malignant phyllodes	Presence of epithelial clefts, with or without CD34 positivity	- Stroma rich malignant PT may have a predominant spindle cell component - CD34 positivity may be helpful if the architecture of PT cannot be identified, although usually negative in high-grade PT. Focal expression of CKs or p63 may be seen in malignant PT but these should not alter the diagnosis if the characteristic architecture is present
Malignant adenomyoepithelioma and myoepithelial carcinomas	Biphasic malignant tumour with expression of both luminal and basal markers	- The malignant spindle cell component of malignant adenomyoepithelioma often displays epithelial with or without myoepithelial/myoid differentiation. This should be considered as MBC arising in adenomyoepithelioma - Pure myoepithelial carcinoma is extremely rare and should show evidence of a residual benign myoepithelial component and predominant myoepithelial immunophenotypic differentiation (Myoepithelial carcinoma is currently classified as MBC for management purposes) - IHC: express CKs and other markers of myoepithelial differentiation including SMA, myosin, p63, CD10, calponin and S100 protein, and ultrastructural evidence of pinocytotic vesicles, myofibrils, patchy basal lamina and tonofilaments
Angiosarcoma	Vaso-formative tumour	- Usually low grade with well-formed anastomosing vascular channels lined by atypical endothelial cells - Spindle cell angiosarcoma is typically of higher grade with poorly defined margins and infiltration of breast parenchyma and fat. May display foci of papillary and solid epithelioid cell proliferation - IHC: positive for CD31, CD34, factor VIII, ERG and D2-40, useful in confirming the diagnosis. CKs may be positive
Melanoma	Melanin pigment deposition, prominent nucleoli. History of melanoma	- Melanoma is one of the most common neoplasms to metastasise to the breast parenchyma - May exhibit any growth pattern including spindle cell sarcomatous, angiosarcomatous, rhabdoid or malignant biphasic pattern. Clinical history and IHC are very useful for its diagnosis - IHC: Positivity for S100 in most cases with or without positivity for melan-A, HMB-45, SOX10 and vimentin
Metastatic sarcomas	History of previous or metastatic sarcoma	- Usually present as one or more circumscribed masses - Clinical history and comparison with the primary tumour together with IHC are most useful for their diagnosis - Examples include metastatic rhabdomyosarcoma, leiomyosarcoma and malignant peripheral nerve sheath tumour (MPNST)

*MBC* metaplastic breast carcinoma, *CK* cytokeratin

**Fig. 8:**
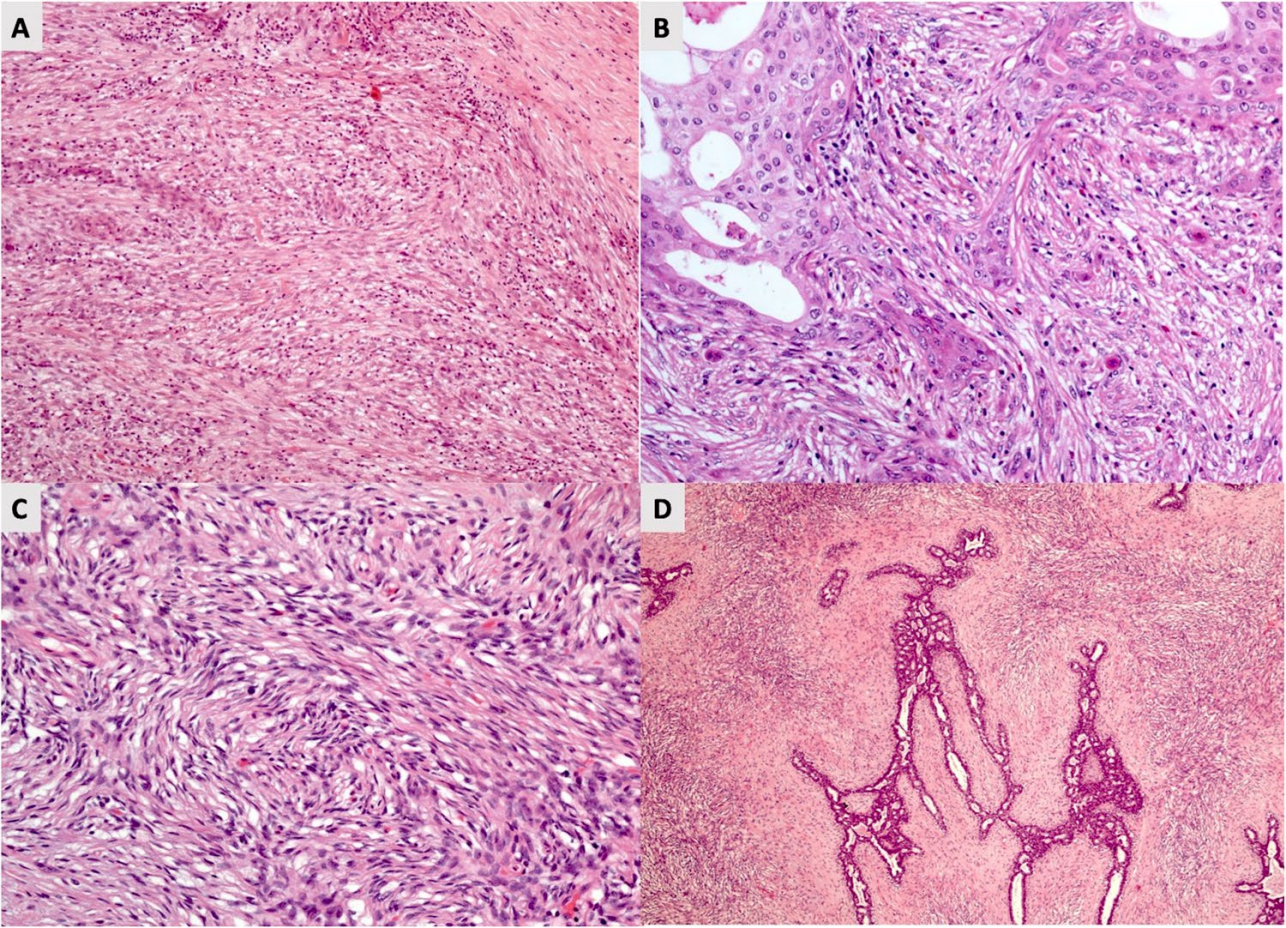
Malignant appearing spindle cell lesion of the breast with no distinguishing features (**A**) and CK negative. However, further sampling revealed areas with squamous cell carcinoma component (**B**) confirming the diagnosis of MBC. **C** shows a case of malignant appearing spindle cell lesion that is negative for CK and CD34. However, further sampling revealed areas with the biphasic growth pattern and characteristic parenchymal component (**D**) confirming the diagnosis of phyllodes tumour

The presence of a recognisable carcinomatous component in mixed spindle cell MBC renders the diagnosis relatively straightforward. In contrast, the diagnosis of pure spindle cell MBC with no obvious morphological evidence of epithelial differentiation is often challenging, even in surgical specimens. Thorough sampling and diligent examination of the histological sections may reveal small foci of in situ or conventional IBC (Fig. 8). Demonstration of epithelial differentiation using CK IHC greatly assists the diagnosis.

The differential diagnosis of a malignant-appearing BSCL includes stroma-rich malignant PT; metastases, most notably metastatic sarcoma, malignant melanoma [11]; lymphoma, primary breast sarcoma, in particular angiosarcoma (Table 4); and benign mimics including florid scar/granulation tissue and nodular fasciitis. The latter two conditions are described above. High-grade malignant myoepithelial BSCLs, such as those arising in adenomyoepithelioma, are rare and frequently show a mixed epithelial and myoepithelial immunophenotype. These are best regarded as MBCs arising in a background of adenomyoepithelioma [52]. Pure malignant spindle cell myoepithelioma with a predominant myoepithelial immunophenotype and a component of benign myoepithelioma is extremely rare.

Stroma-rich malignant PT [53] may present as a pure BSCL particularly on CNB in which only the stromal component is represented. A thorough sampling of excision specimens is advised to identify relevant diagnostic architectural components and to distinguish this entity from spindle cell MBC. The presence of epithelial clefts is pathognomonic of malignant PT. CD34 is a useful marker and although expression is less strong in high grade/malignant PT, the majority of malignant PTs show some degree of CD34 positivity, a feature not seen in MBC. CD10 has been reported in 32–50% of borderline and malignant PTs and its expression has also been reported in some high-grade spindle cell MBCs. [54, 55] It is also important to be aware that, although p63 and CK are frequently expressed by MBC, expression of these markers may be reduced in high-grade tumours and focal expression can be seen in the stroma of some malignant PTs [56, 57].

Metastases to the breast may mimic high-grade spindle cell MBC. The combination of unusual morphology, triple-negative status, and multiple or bilateral lesions is a clue to the diagnosis. Solitary metastases are more likely to be mistaken for high-grade triple-negative spindle cell MBC—the so-called *triple-negative trap*. Tumours that metastasise to the breast that may show spindle cell morphology include leiomyosarcoma and sarcomatoid renal cell carcinoma. Careful evaluation of morphology and clinical history together with judicious use of IHC helps to clarify the diagnosis. Melanoma may assume a spindle cell appearance, but the component cells usually retain some pigment and are positive for the typical IHC markers, melan-A, HMB-45, SOX10, and S100. Lymphoma of the breast may be primary or secondary and unilateral or bilateral. Although more likely to be mistaken for solid invasive lobular carcinoma, it may also resemble spindle cell MBC and should be included in the differential diagnosis. IHC assists diagnosis and accurate subtyping.

Primary breast angiosarcoma, although relatively rare, is the most common primary sarcoma to affect the breast. This may develop spontaneously but is more usually seen in patients who have received radiotherapy to the affected breast. Angiosarcoma of the breast displays a range of morphological appearances ranging from low-grade lesions with well-developed, interconnecting, vascular spaces to poorly differentiated tumours with little or no distinguishing features on H&E. Tumour cells are positive with a range of endothelial markers including CD31, CD34, D2-40, and ERG [58]. CK positivity may be seen in angiosarcoma, mainly in the epithelioid variant [59] which is not included in the differential diagnosis of BSCL. Angiosarcoma is negative for CD10 which may occasionally be seen in MBC. Other primary sarcomas that may affect the breast are exceedingly rare and include leiomyosarcoma, rhabdomyosarcoma, synovial sarcoma, dendritic cell sarcoma, and malignant peripheral nerve sheath tumour. Their histological features and immunoprofiles are similar to those arising at other sites. Apart from the well-established subtypes of sarcoma such as angiosarcoma and malignant PT, several studies have reported a large number of undifferentiated primary breast sarcomas that are purported to arise from the mesenchymal tissue of the breast [9]. These have been variously designated as breast sarcoma, not otherwise specified (currently undifferentiated/unclassified sarcoma), fibrosarcoma, and myxofibrosarcoma [60–62]. Categorisation appears to have been based on lack of expression of epithelial differentiation or other specific markers and are essentially diagnoses of exclusion. In our opinion, most of these tumours are likely to represent poorly differentiated MBC or malignant PT that lacked residual characteristic diagnostic features (see below).

### Categorisation of a malignant SCL with no specific morphological, IHC, or molecular characteristics

Categorisation of a malignant SCL with no specific morphological, IHC, or molecular characteristics of a particular diagnostic entity poses significant challenges for the pathologist particularly with regard to management implications. The current lack of consensus may result in different treatment strategies for patients in different centres. There is accumulating evidence that conventional IBCs may lack expression of one or more CKs or show only very limited expression of a single CK marker [63–65]. These findings support the concept of spindle cell MBC with loss of a range of CKs [10, 40, 66–70]. Some sarcomas also express CKs [71] and accurate diagnosis of these tumours is based on other morphological, IHC and/or molecular features. CK immunoreactivity (positive or negative) is, therefore, not irrefutable evidence in support of a carcinomatous or sarcomatous nature which may represent extremes of a spectrum of differentiation. Furthermore, the diagnostic assays used to characterise these poorly differentiated mesenchymal appearing breast lesions are not entirely specific or sensitive. Molecular assays, including next-generation sequencing (NGS), may help to refine diagnosis but the significance of many of the detected genetic alterations is yet to be confirmed [72]. In practice, it is our approach that, following exclusion of all other possible diagnoses included in the list of differential diagnosis, a CK-negative malignant-appearing BSCL is most appropriately categorised and managed as spindle cell MBC using similar protocols to those used in the treatment of triple-negative IBC-NST [9].

#### Molecular pathology

The pathogenesis of spindle cell MBC and the mechanisms underlying the spindle cell transformation of neoplastic breast epithelial cells are poorly understood. Theories explaining the phenotypic diversity of MBC include a hypothesis that the initial oncogenic events occur in a multipotent progenitor or stem cell with myoepithelial characteristics from which these tumours develop [73]. This is supported by the morphological appearances of spindle cell MBC [74, 75], the expression of several myoepithelial-associated markers [68] and the results of gene expression profiling (GEP) studies [76]. Others have hypothesized that oncogenic events transform normal breast epithelial cells into primitive carcinoma cells that undergo epithelial–mesenchymal transition (EMT). This results in the transformation of an epithelial/carcinomatous tumour into a sarcomatous/spindle cell tumour, giving MBC its characteristic mesenchymal-like morphology [77]. The EMT theory has also been supported by GEP studies of MBC, which have reported de-regulation of genes encoding proteins of cellular adhesion, motility, migration, and extracellular matrix formation [78]. Gene signatures reflecting EMT include upregulation of the EMT triggers Snail, Twist, and transforming growth factor-β, and downregulation of E-cadherin, the salient feature reflecting EMT occurrence [79]. In line with this finding, it was reported that E-cadherin loss had an inverse relationship with nuclear Snail expression in metaplastic chondroid cells in a series of MBCs [80]. In addition, IHC for Snail has been reported as a sensitive, but not specific, diagnostic marker of MBC, as it is also seen in other BSCLs, including PT and myofibroblastoma [81]. Currently, data supporting both hypotheses exist and continue to evolve, and there is no consensus regarding the underlying pathogenesis of MBC. Microarray-based GEP studies have demonstrated that MBC is part of basal-like triple-negative IBC-NST at the transcriptome level [76]. In MBC, identical *p53* mutations were identified in the morphologically distinct carcinomatous and sarcomatous components, suggesting that *p53* mutation is an early event and is maintained during tumour progression, supporting the monoclonal origin of both components [82, 83]. Epidermal growth factor receptor (EGFR) overexpression has been reported in up to 80% of MBCs, with substantial proportions of these cases showing *EGFR* amplification [84, 85]. Although the mechanisms for EGFR overexpression are largely unknown [86], and no activating mutations in *EGFR* have been found in MBC [84], Gilbert et al. demonstrated that a high copy number of *EGFR* is primarily attributable to aneusomy, which was particularly found in tumours with spindle cell or squamous differentiation [87].

Molecular studies of PT, including gene sequencing analysis, showed *MED12* exon 2 somatic mutations in the stroma of borderline and malignant PTs in 65% and 40% respectively [88–92]. Other *MED12* wild-type malignant PTs likely possess alternative driver mutations. There is also evidence that de novo malignant PT may develop through the acquisition of genetic alterations targeting other cancer genes. Somatic mutations in *PIK3CA*,* RARA*, *FLNA*, *SETD2*, and *KMT2D* have been identified in PTs, with borderline and malignant lesions acquiring additional aberrations in cancer-associated genes such as *TP53*, *RB1*, *EGFR*, and *NF1* [91–94]*.* Regions of intra-tumoural heterogeneity within malignant PT appear to exhibit increasing numbers of mutations in parallel *with* morphological attributes of increasing cellularity and nuclear pleomorphism. Recent work has correlated prevalence of *TERT* promoter mutations with increasing malignancy in fibro-epithelial lesions, suggesting a mechanistic role for *TERT* alterations in the progression of these tumours [94–97]. *RARA* mutations, albeit in lower frequency, are also often found in PTs [93]. In a recent targeted deep sequencing and copy number variation (CNV) analysis of recurrently mutated genes in PT [98], five of the six pure primary breast sarcomas (non-angiosarcoma) (83%) cases showed genetic alterations in the TERT gene, mutations in the *MED12* gene (67%), *BCOR* gene (67%), and *KMT2D* gene (50%) in contrast to primary breast angiosarcomas (0% had mutations or copy number alterations in these genes).

## General consideration in the assessment of BSCLs

A thorough approach to the assessment of BSCLs incorporates consideration of the clinical presentation and any relevant clinical history, knowledge of the radiological findings, careful appraisal of morphology (current and previous if relevant), and judicious use of IHC and molecular testing while maintaining a broad differential diagnosis. For example, a history of a rapidly growing nodule in superficial breast tissue together with tissue culture like appearances and extravasation of red blood cells (RBCs) should lead to consideration of a diagnosis of nodular fasciitis. IHC may be used to confirm the diagnosis and to exclude MBC. Nuclear β-catenin positivity in lesional BSCL cells should not be considered pathognomonic of fibromatosis unless fibromatosis-like MBC has been excluded by confirming lack of expression of CKs and p63. In malignant-appearing BSCL, a history of conventional-type IBC treated with radiotherapy raises the possibility of post-irradiation primary breast sarcoma. However, if the prior BC in the affected or contralateral breast was a spindle cell MBC or included a spindle cell component, the lesion is likely to represent a recurrence. In difficult cases, IHC will usually clarify the diagnosis. Osseous or chondrosarcomatous differentiation is more likely to be a component of malignant PT or MBC with heterologous elements rather than a primary osteosarcoma or a chondrosarcoma of the breast [99]. A previous history of soft tissue sarcoma, metastatic sarcomatoid tumour, including sarcomatoid renal cell or squamous cell carcinoma, and melanoma [100] may help to narrow the differential diagnosis.

## Diagnosis of BSCLs on core needle biopsy

Benign-appearing SCLs with clear evidence of CK and p63 expression should be considered malignant (fibromatosis-like MBC) and a B5b diagnosis should be rendered, which necessitates complete excision with sentinel node biopsy. The UK NHSBSP and RCPath guidelines recommend categorising benign-appearing BSCLs that lack defining features on morphology or IHC on CNB as B3 (lesions of uncertain malignant potential [101–103]). This is generally accompanied by a recommendation for further tissue sampling or excision for a definite diagnosis and to exclude the possibility of fibromatosis-like MBC. However, if the diagnosis of fibromatosis is confirmed on CNB, diagnostic biopsy/excision is discouraged to avoid leaving lesional tissue behind as multiple surgical interventions are associated with a higher recurrence rate (see above). A conservative approach or therapeutic excision (excision with margins) is recommended [13, 14, 104]. Myofibroblastoma, nodular fasciitis, and SFT are benign lesions which may be difficult to definitively diagnose on CNB and a B3 categorisation is also recommended [101, 102]. In our experience, scar is the most common BSCL encountered on CNB. Diagnosis is often straightforward, assisted by the presence of inflammation, haemosiderin-laden macrophages or fat necrosis and the lesion can be categorized as B2 (benign) [101]. In mature scars, these features may be absent, and the differential diagnosis may include fibromatosis and other benign lesions. Clinical history and correlation with radiology may be helpful but, in doubtful cases, a B3 designation may be most appropriate. Distinguishing fibrous tissue reaction related to the previous CNB from the actual BSCL in the excision specimen can be facilitated by reviewing the CNB slides.

At the malignant end of the spectrum, spindle cell MBC is the most common diagnosis. A malignant diagnosis (B5b [101]) can be made if the spindle cell lesion shows definite (significant) atypia and/or evidence of epithelial differentiation on morphology or IHC. However, focal epithelial differentiation based purely on IHC should be interpreted with caution and a broad differential diagnosis, including malignant PT and metastases, considered. The categorisation of malignant PT and metastatic tumours in CNB is not well defined. Some authors suggested a B5d category for such malignant tumours [105] but this is not widely accepted and a B5b category with free text explanation in the report is recommended. The IHC panel in such cases should include epithelial differentiation markers, including multiple low and high molecular weight CKs, and other diagnostic markers such as CD34 and S100 to assist the exclusion of other entities. Occasionally, the features are clearly malignant, but a specific diagnosis is not possible. In such cases, the difficulty in categorisation should be stated in the report and a definite diagnosis should be attempted on the surgical specimen. The opinion of a specialist soft tissue pathologist may also be helpful.

*In conclusion*, BSCLs constitute a broad spectrum of morphologically overlapping entities, ranging from benign/reactive processes to aggressive malignant tumours with different management strategies. BSCLs can generally be classified as bland-appearing and malignant-appearing lesions. In addition to the morphology, clinical history and IHC are most helpful in their diagnosis. Molecular assays may help in the diagnosis of some lesions, but they are not available in most laboratories. It is important to consider a wide differential diagnosis and to have a structured approach with focussed IHC panels. It is essential to distinguish low-grade spindle cell MBC from other “benign” entities in view of the prognostic and management implications. Many BSCLs are diagnosed as B3 on CNB and require careful multidisciplinary review.
